# Ubiquitinated AIF is a major mediator of hypoxia-induced mitochondrial dysfunction and pulmonary artery smooth muscle cell proliferation

**DOI:** 10.1186/s13578-022-00744-3

**Published:** 2022-01-28

**Authors:** Cui Ma, Xiaoying Wang, Siyu He, Lixin Zhang, June Bai, Lihui Qu, Jing Qi, Xiaodong Zheng, Xiangrui Zhu, Jian Mei, Xiaoyu Guan, Hao Yuan, Daling Zhu

**Affiliations:** 1 Central Laboratory of Harbin Medical University (Daqing), 39 Xinyang Road, Daqing, 163319 People’s Republic of China; 2 College of Medical Laboratory Science and Technology, Harbin Medical University (Daqing), Daqing, 163319 People’s Republic of China; 3 College of Pharmacy, Harbin Medical University, Harbin, 150081 People’s Republic of China; 4 College of Basic Medical Sciences, Harbin Medical University (Daqing), Daqing, 163319 People’s Republic of China; 5 State Province Key Laboratories of Biomedicine-Pharmaceutics of China, Daqing, 163319 People’s Republic of China; 6 Key Laboratory of Cardiovascular Medicine Research, Ministry of Education, Daqing, 163319 People’s Republic of China

**Keywords:** AIF, Pulmonary artery smooth muscle cells, Mitochondria, Mitophagy and autophagy

## Abstract

**Background:**

Excessive proliferation of pulmonary artery smooth muscle cells (PASMCs) is the main cause of hypoxic pulmonary hypertension (PH), and mitochondrial homeostasis plays a crucial role. However, the specific molecular regulatory mechanism of mitochondrial function in PASMCs remains unclear.

**Methods:**

In this study, using the CCK8 assay, EdU incorporation, flow cytometry, Western blotting, co-IP, mass spectrometry, electron microscopy, immunofluorescence, Seahorse extracellular flux analysis and echocardiography, we investigated the specific involvement of apoptosis-inducing factor (AIF), a mitochondrial oxidoreductase in regulating mitochondrial energy metabolism and mitophagy in PASMCs.

**Results:**

In vitro, AIF deficiency in hypoxia leads to impaired oxidative phosphorylation and increased glycolysis and ROS release because of the loss of mitochondrial complex I activity. AIF was also downregulated and ubiquitinated under hypoxia leading to the abnormal occurrence of mitophagy and autophagy through its interaction with ubiquitin protein UBA52. In vivo, treatment with the adeno-associated virus vector to overexpress AIF protected pulmonary vascular remodeling from dysfunctional and abnormal proliferation.

**Conclusions:**

Taken together, our results identify AIF as a potential therapeutic target for PH and reveal a novel posttranscriptional regulatory mechanism in hypoxia-induced mitochondrial dysfunction.

**Supplementary Information:**

The online version contains supplementary material available at 10.1186/s13578-022-00744-3.

## Introduction

Pulmonary hypertension (PH) is a fatal cardiovascular and respiratory system disease characterized by a progressive increase in the mean pulmonary artery pressure and symptoms of exertional dyspnea, resulting in right ventricular failure and death [1, 2]. Hypoxia is considered as a common cause of pulmonary vasculature pathological changes in PH that induce pulmonary vascular remodeling by regulating various cellular processes, such as cell proliferation or differentiation [3], pyroptosis [4], autophagy [5], and pulmonary vascular calcification [6]. Among these processes, the hyperproliferation of pulmonary artery smooth muscle cells (PASMCs) is the key influencer of pulmonary vascular remodeling. However, the exact molecular and cellular mechanisms regulating the pathological process of pulmonary vascular remodeling remain poorly understood.

Mitochondria are dynamic, double-membrane-bound organelles [7] that play various regulatory roles in calcium signal transduction [8], phospholipid metabolism [9], apoptosis [10], reactive oxygen species (ROS) production and iron sulfur biosynthesis [11, 12]. Mitochondria, as the essential organelles for eukaryotic life, produce 90% of the energy or adenosine triphosphate (ATP) required by the cell and are called as the powerhouses of the cell [11, 13]. The mitochondrial respiratory chain is called the “production line” of mitochondrial energy provision, which is the primary production pathway for ATP and comprises five linked membrane protein complexes, termed complexes I, II, III, IV and V [14, 15]. When the function of the mitochondrial respiratory chain is impaired, the electron transport chain is uncoupled from ATP, leading to ROS production, which destroys mitochondrial structure and function, causing many diseases [16, 17]. For example, the abnormal expression of mitochondrial complex I impairs the mitochondrial respiratory chain, resulting in increased hepatic glucose output, which leads to diabetes [18]. The inactivity of mitochondrial complex III leads to mitochondrial respiratory chain dysfunction, reduces ATP and elevates ROS production, which promotes the development of Parkinson’s disease [19]. The decrease in mitochondrial respiratory chain activity increases of ROS generation and cardiolipin oxidation, causing mitochondrial dysfunction, which further aggravates myocardial ischemia reperfusion injury [20]. These findings highlight that mitochondrial homeostasis is crucial in the progression of various diseases.

Mitochondrial autophagy (mitophagy) is a selective autophagy that removes damaged and dysfunctional mitochondria through the autophagy lysosome pathway to maintain a certain number of mitochondria in cells, ensuring the energy supply and normal function of cells [15, 21, 22]. Mitophagy plays a role in several diseases. For example, adenosine monophosphate-activated protein kinase (AMPK) activates Pink/Parkin-mediated mitochondrial autophagy through Pink phosphorylation, effectively preventing heart failure [23]. Macrophage stimulator 1 regulates Parkin-dependent mitophagy through the AMPK pathway, inhibiting the apoptosis of liver mitochondria and consequently promoting the development of nonalcoholic fatty liver disease [24]. Mitophagy mediated by Pink/Parkin activates AMPKα phosphorylation to boost vascular smooth muscle cell proliferation by inducing apelin-13, which aggravates the progression of atherosclerosis [25]. However, the pathophysiological mechanisms of mitophagy remain unclear, particularly in hypoxic PH.

Apoptosis-inducing factor (AIF) is a mitochondrial intermembrane space flavoprotein encoded by the X-chromosome Aifm1 (Pdcd8) gene that regulates cell survival and death [26, 27]. AIF is generated in the cytoplasm and enters the mitochondrial intermembrane space to perpetuate mitochondrial morphology and crista structure [28]. The loss of AIF destroys the structure and function of mitochondria and reduces the content of respiratory chain protein complexes, disrupting oxidative phosphorylation (OXPHOS) and causing mitochondrial disorders [29, 30]. Presently, many reports have shown that AIF plays important roles in many diseases by participating in mitochondrial metabolism. For example, AIF inhibits the oxidation of phosphatase and tensin homolog on chromosome ten (PTEN) by directly binding to it, slowing tumor progression [31]. In anorexigenic proopiomelanocortin (POMC) neurons, AIF inhibition results in partial damage to mitochondrial OXPHOS and enhances the availability of fatty acids and ROS formation, which ameliorate systemic glucose metabolism in obesity [32]. Inactivation of AIF in the heart results in dilated cardiomyopathy by disturbing the function and abundance of protein subunits of complex I [33]. However, whether AIF is implicated in hypoxia-induced mitochondrial energy metabolism and mitophagy in PH is unclear.

Therefore, this study aimed to identify the role of AIF in the development of hypoxia-induced PH by regulating mitochondrial function. We found for the first time that ubiquitinated and decreased AIF expression induced by hypoxia resulted in mitochondrial respiratory chain impairment with abnormal mitochondrial metabolism, elevated mitophagy and autophagy, and eventually increased cell proliferation.

## Materials and methods

### Antibodies and reagents

Antibodies against AIF, UBA52, Pink, Parkin, Tom20, Lamp2a, LC3B and α-SMA (Catalog numbers ab32516, ab109227, ab23707, ab77924, ab56783, ab18528, ab48394 and ab7817) were obtained from Abcam (Cambridge, UK). Antibodies against HK II, PKM2 and PDH (Catalog numbers 2867, 4053 and 3205) were obtained from Cell Signaling Technology (Danvers, MA, US). Antibodies against Cyclin A, Cyclin D, PCNA, UB, P62, ATG5 and ATG7 (Catalog numbers BM1582, BM4272, BM0104, BM4359, BA2849, BA3525 and BA3527) were obtained from Boster (Wuhan, China). Bortezomib (PS-341) and MG132 (Catalog numbers S1013 and S2619) were obtained from Selleck Chemicals (Pittsburgh, PA, US).

### Animals use

Rats (Sprague–Dawley, male, 6–9 weeks old) and mice (C57BL/6, male, 6–9 weeks old) were obtained from the Experimental Animal Center of Harbin Medical University. The corresponding target RNA cloning construction and serotype 5 adenovirus-associated virus (AAV 5) packaging experiment were completed by Genechem (Shanghai, China). Mice weighing approximately 25 g each were randomly divided into different groups. Mice were infected with the AAV 5 vector at 10^11^ genome equivalents in 30 µl HBSS (Hanks’ Balanced Salt Solution) after isoflurane anesthesia and followed by nasal drops. Next, mice were treated in normoxic and hypoxic environments for 28 days with inspired oxygen (FiO_2_) fractions of 0.21 and 0.12 respectively, as previously described [34]. The concentration of oxygen was monitored continuously using an oxygen analyzer (P110, BioSpherix New York, US). The surgical procedure was performed under avertin (200 mg/kg i.p., Sigma-Aldrich, St Louis, USA) anesthesia and pain was minimized.

### Echocardiography, right ventricular systolic pressure (RVSP) and ventricular hypertrophy measurements

At the end of hypoxic treatment, mice were anesthetized and undergoing echocardiography using a Vevo2100 imaging system (VisualSonicse Inc., Toronto, Ontario, Canada) with a 37 mHZ probe. Stable images were obtained in the M, B and Doppler modes, and the pulmonary artery velocity time integral (PAVTI), pulmonary artery acceleration time (PAAT) and left ventricular ejection fraction (LVEF) were measured. The RVSP was measured with PowerLab monitoring equipment (AD Instruments, Colorado Springs, Colo). Briefly, a 1.2 French Pressure Catheter (Scisense Inc.) was connected to the Scisense FA-404 recorder. The catheter was inserted into the superior vena cava, and finally into the right ventricular vein. RVSP was continuously recorded for 45 min. After measurement of RVSP, heart was dissected and weighed for calculation of the right ventricular hypertrophy index (ratio of right ventricular free wall weight over sum of septum plus left ventricular free wall weight: RV/LV + S).

### Histology and immunohistochemistry

Fresh lung tissues were fixed in 4% paraformaldehyde for 2–3 days. Next, the tissues were dehydrated and embedded in paraffin wax and cut into 5 μm thick sections to stain with hematoxylin and eosin (H&E) or Masson’s trichrome stain as appropriate. For immunohistochemistry, 5 μm thick sections were deparaffinized and rehydrated in alcohol. Antibodies were incubated with α-SMA (1:100), AIF (1:100) and PCNA (1:100). The detailed immunohistochemistry methods were performed according to a technique described previously [35].

### Cell isolation and culture

Pulmonary artery smooth muscle cells (PASMCs) were cultured and isolated according to our previously published protocol [36]. Briefly, pulmonary arteries were quickly isolated from the rat lungs and then digested with PBS solution containing 0.2% collagenase and 0.2% bovine serum albumin at 37 °C for 3 h. The digested PASMCs were cultured in DMEM containing 20% fetal bovine serum (FBS) for 3 days in a humidified incubator with 5% CO_2_ at 37 °C. Passages 2–5 of PASMCs were used for subsequent experiments before cultured in serum-free DMEM medium for 12–24 h. Tri-Gas incubator (Thermo Fisher) with an atmosphere comprising 3% O_2_, 5% CO_2_ and 92% N_2_ was used for hypoxic culture.

### Immunofluorescence

PASMCs were fixed with 4% paraformaldehyde for 15 min and permeabilized with 0.01% Triton X-100 for 15 min at room temperature. Next, 5% Bovine Serum Albumin was used to block the cells at room temperature for 30 min, and then the cells were incubated with antibodies against AIF (1:50), Tom20 (1:100), UB (1:50) and Lamp2a (1:100) at 4 °C overnight. The cells were washed three times with PBS, and incubated with Cy3/FITC-conjugated secondary antibody (1:100) at 37 °C for 2 h and DAPI away from light for 10 min. The results of immunofluorescence staining were recorded by a living cell workstation (AF6000; Leica).

### Transmission electron microscopy (TEM)

Cultured PASMCs from control and hypoxic conditions were processed for ultrastructural analysis by TEM. Samples were fixed in 2% glutaraldehyde in 0.1 M sodium cacodylate buffer (pH 7.2) and were post fixed in 1% osmium tetroxide containing 1.5% potassium cyanoferrate for 2 h at 4 °C. After contrasted with uranyl acetate 2% in water and gradually dehydrated in ethanol (30 to 100%), ultrathin sections (50 nm) were stained with uranyl acetate and lead citrate and then examined under a TEM (Hitachi-7650, Japan).

### Plasmid construction, siRNA design and transfection

AIF overexpression plasmid was constructed using the GV141 vector by GeneChem (Shanghai, China). The vector alone was used as a negative control (NC). The small interfering RNA against AIF and UBA52 was designed and synthesized by GenePharma (Suzhou, China). Non-targeted control siRNA (siNC) was used as negative control. Transfection was performed according to the manufacturer’s instructions for the Lipofectamine^®^ 2000 Reagent (Life Technologies, Carlsbad, USA). PASMCs were cultured to 70% confluence, and 3 ug plasmids/NC or 2 µg siRNA/NC and 10 µl of transfection reagent were mixed in serum-free DMEM medium and then added it directly to the cells. The cells were then incubated for 24–48 h to be used as required. The sequences are listed below:

siNC:

sense, 5′-UUCUCCGAACGUGUCACGUTT-3′,

antisense, 5′-ACGUGACACGUUCGGAGAATT-3′.

siAIF:

sense, 5′-CCUCAGGCAUAGAAGUGAUTT- 3′,

antisense, 5′-AUCACUUCUAUGCCUGAGGTT-3′.

siUBA52:

sense, 5′-GAAGUACAACUGUGACAAGAUTT- 3′,

antisense, 5′-AUCUUGUCACAGUUGUACUUCTT-3′.

### Western blot analysis

Lung tissues and PASMCs were broken by sonication on ice, then centrifuged at 13,500 rpm for 20 min at 4 °C to collect supernatants and stored at − 80 °C for use in Western blot analysis. Protein samples (30 µg) were separated on a 10% SDS-PAGE gel and transferred onto the nitrocellulose membranes. The protein-adhered membranes were blocked with 5% nonfat dry milk for 1 h and incubated with specific antibodies against AIF (1:1000), HK II (1:1000), PKM2 (1:1000), PDH (1:1000), Pink (1:1000), Parkin (1:1000), ATG5 (1:500), ATG7 (1:500), P62 (1:500), LC3B (1:4000), UBA52 (1:1000), PCNA (1:500), Cyclin A (1:500), Cyclin D (1:500) and Actin (1:500). Next, the protein-adhered membranes were reacted with horseradish peroxidase-conjugated secondary antibodies and subjected to chemiluminescence reagent imaging.

### Quantitative RT-PCR

Total RNA was isolated from lung tissue and cultured PASMCs using TRIzol reagent according to the manufacturer’s protocol (Invitrogen, Carlsbad, USA). Next, the extracted RNA was reverse transcribed into cDNA according to the manufacturer’s protocol in a reverse transcription kit. Using a Roche Light Cycler 480II, real-time PCR was performed with SYBR Green (TOYOBO, Tokyo, Japan). β-Actin was used as the internal control. The sequences of PCR primers were as follows:

AIF (Rat):

forward, 5′-AAGAATAATGGGATTAGGAC-3′,

reverse, 5′-AAGGGACGTGACTTGGTA-3′.

AIF (mouse):

forward, 5′-CAACTCCTACTGCTCCTTCTAAC-3′,

reverse, 5′-CCACTGTTTTCCAAATCACG-3′.

β-Actin (Rat):

forward, 5′-AGGGAAATCGTGCGTGAC-3′,

reverse, 5′-CAAGAAGGAAGGCTGGAAAA-3′.

β-Actin (mouse):

forward, 5′-TCAGGTCATCACTATCGGCAAT-3′,

reverse, 5′-AAAGAAAGGGTGTAAAACGCA-3′.

### Mitochondrial oxidative phosphorylation (OXPHOS) and glycolysis assays

The oxygen consumption rate (OCR, indicating of mitochondrial OXPHOS) and extracellular acidification rate (ECAR, indicating of glycolysis) were measured using an extracellular flux analyzer (Seahorse Bioscience, Billerica, MA, USA). For OCR analysis, PASMCs were seeded into 24-well Seahorse XF plates at 4 × 10^4^ cells/well, 5 mM glucose and 2 mM sodium pyruvate were then added. The plates were incubated in a CO_2_-free XF prep station at 37 °C for 40 min to allow temperature and pH calibration. After that, we sequentially injected the ATP synthase inhibitor oligomycin (1 μM), which decreases OCR levels, followed by the electron transport chain accelerator FCCP (1 μM), which causes maximal respiration and finally rotenone + antimycin A (both 1 μM), which are mitochondrial complex I and III inhibitors, respectively. Then, the mitochondrial respiratory parameters were calculated from the OCR values in picomoles per minute of oxygen consumed. For the glycolysis assay, PASMCs were glucose starved in XF assay medium in a CO_2_-free XF prep station and then treated with glucose (2 mg/ml), oligomycin (1 μM) and 2-deoxy-d-glucose (100 mM). Differences in the basal, maximal and spare glycolytic capacity were represented as mpH/min.

### Cell counting kit 8 (CCK8) assay

PASMCs were seeded in 96-well plates and treated according to the different experimental groups within hypoxic environment (3% O_2_). After cultured for 24 h, CCK8 reagent (10 µl) was added to each well with incubation at 37 °C for 2 h. Then, the results were detected at 450 nm wavelength using a spectrophotometer.

### EdU incorporation assay

PASMCs in different groups were treated with 50 μmol/l of 5-ethynyl-2ʹ-deoxyuridine (EdU, RiboBio, Guangzhou, China) and incubated for 4 h at 37 °C. The cells were fixed with 4% formaldehyde for 20 min and exposed to 0.5% Triton X-100 for 15 min. The cells were washed with PBS for 3 times, and then reacted with 100 μl of Apollo^®^ reaction cocktail for half an hour. Next, DAPI (5 μg/ml) was used to stain the DNA of cells in each well for 30 min, and then the cells were observed under a fluorescence microscope. The results were expressed as the percentage of proliferating cells (EdU-positive cells) with DAPI and were quantitated using Image-Pro Plus.

### Cell cycle analysis

PASMCs were subjected to different reagents, and then cell precipitates were collected and resuspended in 1 ml of PBS. Subsequently, different groups of cells were fixed with 70% ethanol and were exposed to 500 µl of propidium iodide at 37 °C for 20 min. A BD FACSCalibur flow cytometer was used to measure DNA fluorescence. For each cell group, 2 × 10^4^ cell events were accumulated in a histogram. The proportions of cells in the different phases of the cell cycle were calculated from each histogram.

### Coimmunoprecipitation

PASMCs were washed three times with cold PBS, and then 1 ml of RIPA lysis buffer plus PMSF (1:100) was added to homogenize the cells. The cell lysis solution was collected and incubated with slow shaking on a shaking platform for 30 min at 4 °C. Next, the cell lysis solution was centrifuged at 15,000 rpm for 30 min at 4 °C to collect the supernatant followed by adding 5 μg of target antibody or IgG and incubating at 4 °C for 6 h. After that, Protein A + G agarose beads were added overnight in a 4 °C shaker. The next day, PBS was used to wash the antibody-protein complexes, and then the pellet was collected and resuspended in protein loading buffer (2×). The samples were then subjected to western blot or mass spectrometry analysis. The mass spectrometry analysis was performed by Beijing Bio-Tech Pack Technology Company Ltd.

### Autophagic flux monitoring assay

To monitor cell autophagic flux, PASMCs were cultured on coverslips and transfected with tandem ad-mCherry-GFP-LC3B adenovirus (C3011, Beyotime, Shanghai, China) for 48 h at a multiplicity of infection (MOI) of 20. For mitophagy, cells were transfected with 1 µg pMitophagy Keima-Red mPark2 plasmid (AM-V0259M, MBL, Janpan). Next, the experimental procedure was carried out according to the manufacturer’s instructions, GFP and red puncta were captured under a fluorescence microscope.

### MitoSOX

Mitochondrial ROS activity was measured with MitoSOX Red (M36008, Invitrogen, Carlsbad, USA), a redox-sensitive fluorescent probe that is selectively targeted to the mitochondria. PASMCs were incubated with 5 µM MitoSOX probe for 20 min. The cells were washed with PBS, and red fluorescence was captured (514 nm excitation/585 nm emission) from ≥ 3 optical fields. Mitochondrial ROS was quantified by Image J in fluorescence intensity of treated cells.

### Mitochondrial complex activity assay

The activities of mitochondrial respiratory chain complex I–V were detected according to the instructions of Micro Mitochondrial Respiratory Chain Complex I–V Activity Assay Kit respectively (Solarbio Life Sciences, Beijing, China). The activity of mitochondrial respiratory chain complex I (decrease in nicotinamide adenine dinucleotide) was determined at 340 nm, complex II (decrease in 2,6-dichloroindophenol) was determined at 605 nm, complex III (reductive cytochrome *C*) and IV (decrease in reduced cytochrome *C*) was determined at 550 nm, and complex V (increase in phosphate) was determined at 660 nm by spectrophotomer (SpectraMax 190, Molecular Devices, US).

### Statistical analysis

Statistical analysis was performed using GraphPad Prism 8 (GraphPad Software, LaJolla, CA, USA) software. Statistical analysis was performed using Student’s t-test or one-way ANOVA followed by Dunnett’s test where appropriate. p < 0.05 was considered statistically significant.

## Results

### Hypoxia induces mitochondrial complex I lesions and AIF downregulation

**Fig. 1:**
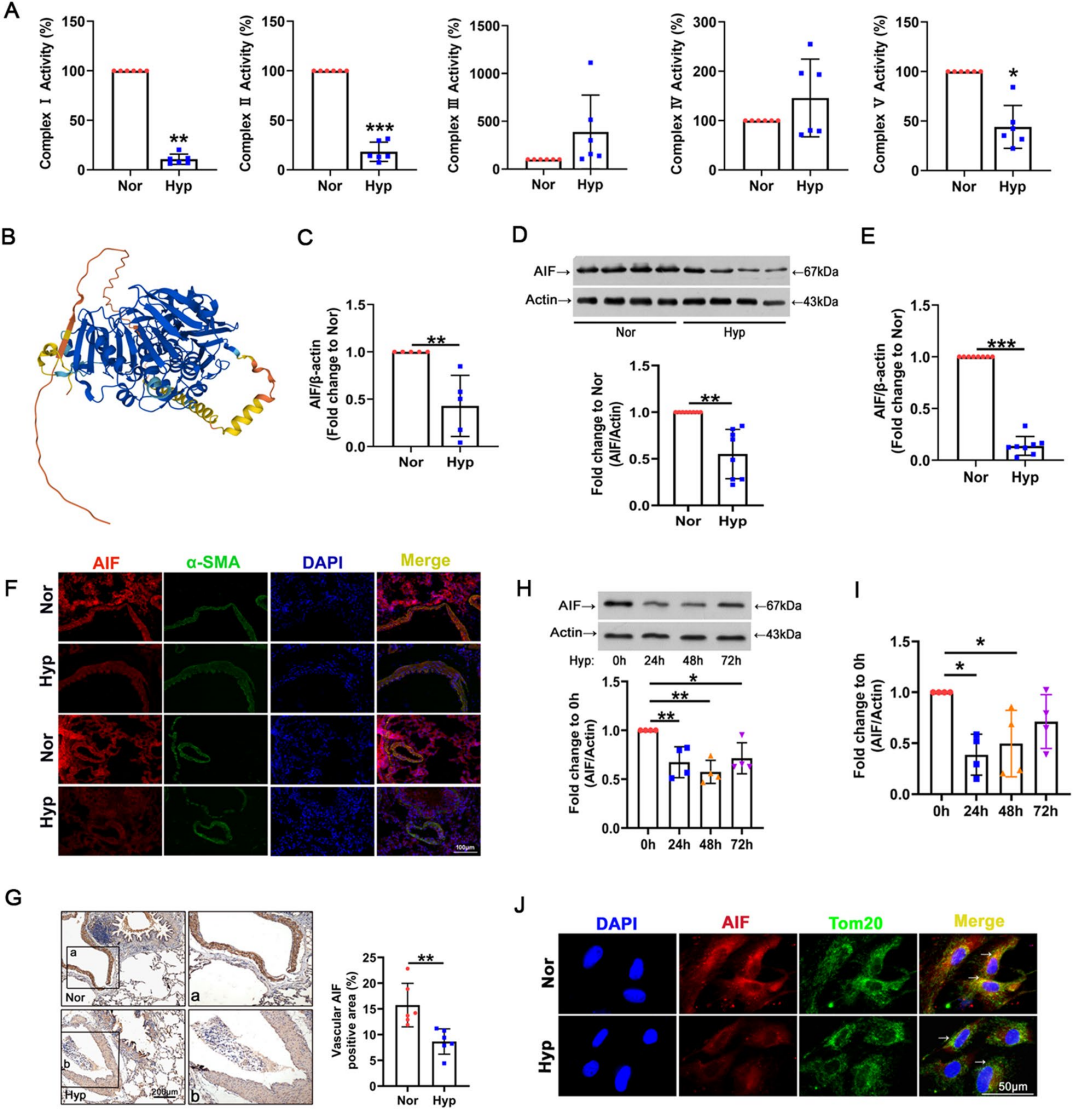
Hypoxia results in decreased AIF expression. **A** Activity of mitochondrial respiratory chain complexes I–V after exposure to hypoxia, with complex I showing the most severe damage (n = 6). **B** Schematic structural model of AIF protein. **C** Decreased AIF expression was found in plasma from the hypoxic group (n = 5). **D**, **E** AIF protein and RNA levels in the lung tissues of the hypoxic model (n = 8). **F**, **G** The location of AIF in the smooth muscle layer of lung tissues from the hypoxic model was determined by immunofluorescence (**F**, Scale bar = 100 μm) and immunohistochemical staining analysis (**G**, Scale bar = 200 μm) (n = 3). **H**, **I** Time course of AIF protein and RNA levels in PASMCs 0, 24, 48, and 72 h after hypoxia treatment. **J** Subcellular distribution of AIF in PASMCs as determined by immunofluorescence analysis. Scale bars: 50 μm (n = 3). All data are presented as the means ± standard deviation. *p < 0.05; **p < 0.01; ***p < 0.001; *Nor* normoxia, *Hyp* hypoxia

To verify mitochondrial dysfunction in hypoxia, we first assessed the activities of mitochondrial respiratory chain complexes in lung tissue. As shown in Fig. 1A, hypoxia exposure significantly reduced the activity of complexes I, II and V, and no obvious changes were observed in the activities of mitochondrial complexes III and IV. The finding that the activity of complex 1 showed the most obvious decrease led us to investigate the mechanisms for regulating complex I injury in hypoxia. AIF shares homology with yeast Ndi1, a NADH oxidoreductase that reduces mitochondrial damage and complex I lesions [37]. The structure of AIF from UniProtKB-Q9JM53 (https://www.uniprot.org/) is shown in Fig. 1B. To determine the role of AIF in damage to the mitochondrial respiratory chain and hypoxic PH, we first investigated the expression and location of AIF in an animal model of hypoxia. AIF expression was decreased in plasma from the hypoxic group, as revealed by qRT-PCR (Fig. 1C). Reduced protein and mRNA levels of AIF were also found in hypoxic lung tissue by Western blotting and qRT-PCR (Fig. 1D, E), and the smooth muscle layer of the vascular media was the main location of AIF (Fig. 1F, G). Consequently, the deficient AIF expression was confirmed at the posttranslational and transcriptional levels in PASMCs exposed to hypoxia (Fig. 1H, I). We then performed fluorescence analysis to determine the cellular distribution of AIF and found that AIF colocalized with the mitochondrial marker Tom20, indicating that compared with the high AIF expression under normal conditions, AIF expression was decreased under hypoxic conditions in both the mitochondria and cytoplasm (Fig. 1J). These results demonstrate that AIF deregulation in lung tissue and PASMCs may be related to damage to the mitochondrial respiratory chain.

### AIF participates in the hypoxia-induced mitochondrial energy phenotype switch and oxidative stress

**Fig. 2:**
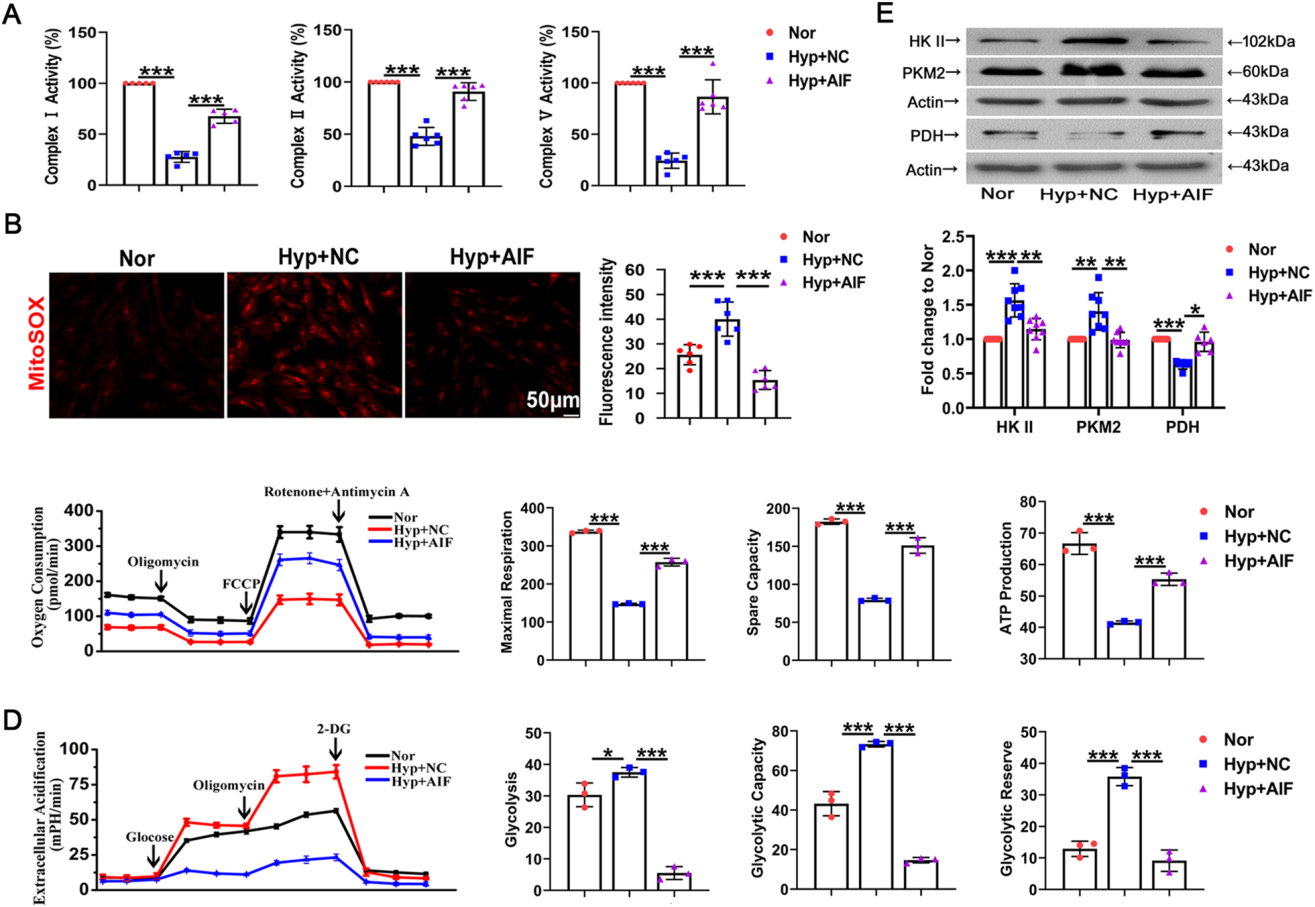
Hypoxic mitochondrial dysfunction in PASMCs is mediated by AIF. **A** Activity of complexes I, II and V after pretreatment with AIF overexpressed plasmid (n = 5–6). **B** Representative images and averaged values of mitochondrial ROS (n = 6). Scale bars: 50 μm. **C** The oxygen consumption rates of cultured rat PASMCs, maximal respiration, spare respiratory capacity and derived mitochondrial ATP production were calculated following oligomycin and FCCP treatment (n = 3). **D** Glycolysis and glycolytic reserve following treatment with 10 mM glucose and 1 μM oligomycin were measured in cultured PASMCs, and nonglycolytic acidification after treatment with 100 mM 2-deoxyglucose was detected (n = 3). **E** Western blot analysis of HK II, PKM2 and PDH protein expression in PASMCs in the presence or absence of hypoxia (n = 6–8). All data are presented as the means ± standard deviation. *p < 0.05; **p < 0.01; ***p < 0.001; *Nor* normoxia, *Hyp* hypoxia, *NC* negative control

To further confirm the roles of AIF in cellular oxidative stress, we used an AIF-overexpressing plasmid to transfect cultured PASMCs. The transfection specificity and efficiency are shown in Additional file 1: Fig. S1A. Next, the activity of mitochondrial complexes I, II and V was measured after exposure to hypoxia in PASMCs. The complex activities were enhanced by AIF overexpression (Fig. 2A). To investigate the role of AIF in mitochondrial oxidative stress injury, we used a mitochondrially targeted superoxide indicator (MitoSOX). MitoSOX fluorescence was increased after 24 h of hypoxia exposure, and treatment with the AIF overexpression plasmid blocked the increase in mitochondrial reactive oxygen species (ROS) (Fig. 2B). The metabolic patterns of mitochondrial aerobic respiration in real time were monitored using a Seahorse XFe24 extracellular flux analyzer. Oxygen consumption rate analysis revealed that hypoxia decreased maximal or spare respiratory capacities and mitochondrial ATP production were relieved by overexpressing AIF in cultured PASMCs (Fig. 2C). Furthermore, the extracellular acidification rates were evaluated to estimate cellular glycolysis. Glycolytic stress activation stimulated by hypoxia was blocked with AIF overexpression (Fig. 2D). The key metabolic enzyme immunoblotting assay indicated that increased expression of glycolytic enzymes, including hexokinase II (HK II) and pyruvate kinase muscle isozyme 2 (PKM2), and decreased oxidative phosphorylation enzyme pyruvate dehydrogenase (PDH) induced by hypoxia were reversed by AIF overexpression (Fig. 2E). These data revealed that hypoxia-induced mitochondrial phenotypic transition and oxidative stress dysfunction were regulated by AIF.

### The role of AIF in mitophagy and autophagy triggered by hypoxia

**Fig. 3:**
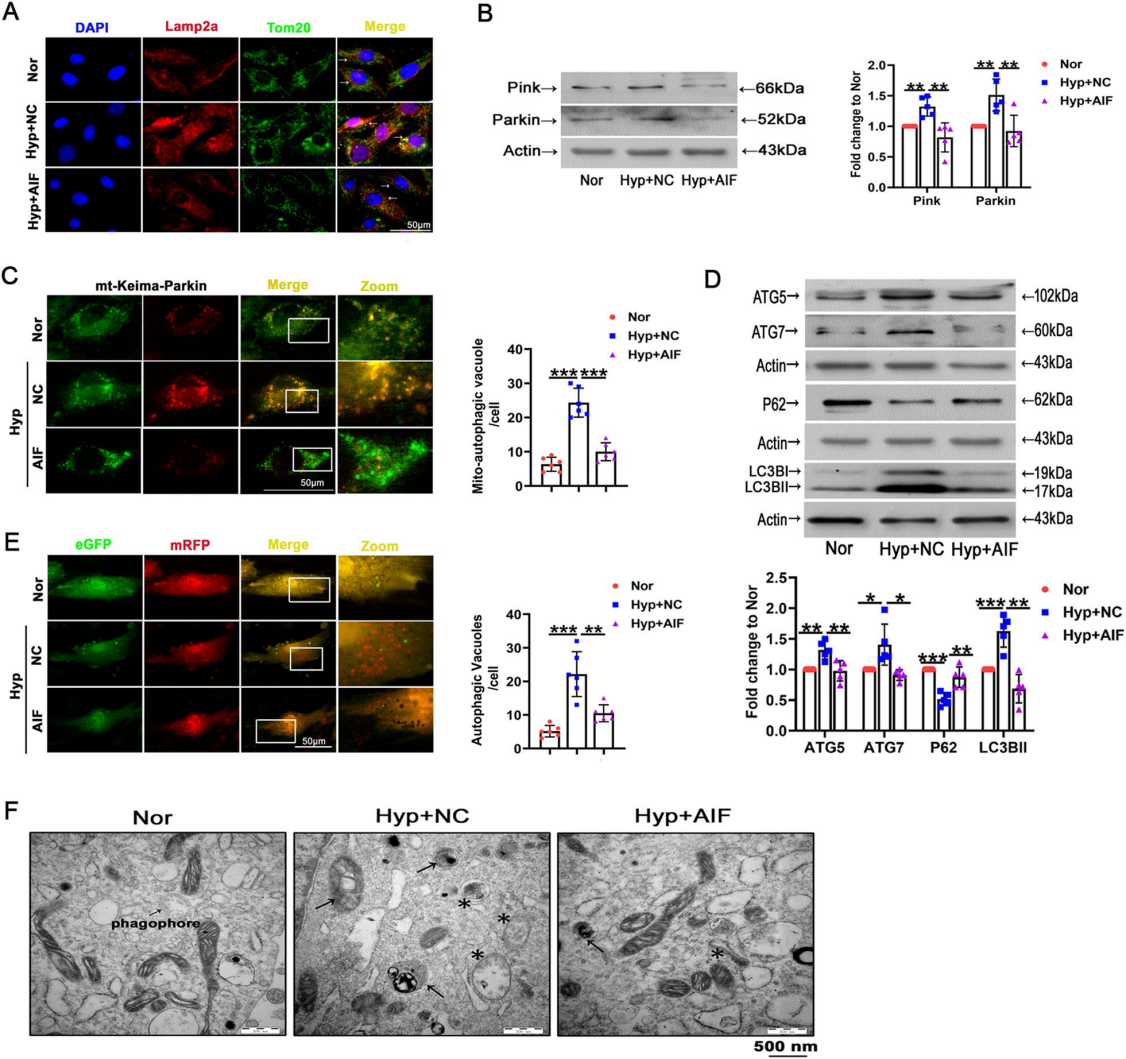
Effects of hypoxia and AIF on mitophagy and autophagy in PASMCs. **A** AIF influenced mitochondrial function by obstructing Tom20 and Lamp2a colocalization, as indicated by the decreased yellow area. Scale bars: 50 μm (n = 4). **B** PASMCs were exposed to hypoxia for 24 h, and the expression of Pink and Parkin was evaluated by Western blotting (n = 5). **C** AIF overexpression mitigated the alteration of mitophagy in PASMCs as determined by Mitophagy Keima-Red plasmid transfection. The number of mitochondrial autophagosomes (yellow dots) was calculated (n = 5). Scale bars: 50 μm. **D** The expression of LC3B-II, P62 and ATG5/7 was evaluated by Western blotting (n = 5). **E** Autophagic flux and the formation of autophagosomes were detected (n = 6). Scale bars: 50 μm. **F** Representative electron micrograph of cells after Nor and Hyp treatment. Asterisks represent autophagosomes and arrows represent autolysosomes. Scale bars: 500 nm (n = 3). All data are presented as the means ± standard deviation. *p < 0.05; **p < 0.01; ***p < 0.001; *Nor* normoxia, *Hyp* hypoxia, *NC* negative control

The above findings showed that AIF downregulation in PASMCs was related to the accumulation of damaged mitochondria, prompting us to investigate the role of AIF in autophagic degradation of mitochondria (mitophagy) in response to hypoxia exposure. Colocalization of Tom20-stained mitochondria and Lamp2a-labeled lysosomes was used to determine mitolysosome formation. As shown in Fig. 3A, hypoxia resulted in increased mitophagy, and the mitolysosome formation was decreased by AIF overexpression. In addition, PASMCs from hypoxia showed greater immunoreactivity with the mitophagy markers Pink and Parkin than normal subjects, and AIF attenuated the increase (Fig. 3B). Hypoxia-induced mitophagy was further confirmed in PASMCs by “pMitophagy Keima-Red mPark2” a pH-sensitive fluorescent protein expression plasmid that monitors mitophagy and contains parkin, which is essential for the induction of mitophagy. The excitation spectrum of Keima shifts from 458 nm (green; mitochondria at neutral pH) to 561 nm (red; mitochondria under acidic pH) when mitochondria are delivered to acidic lysosomes [38, 39]. Compared with normoxia, hypoxia treatment induced mitochondrial delivery to lysosomes, an effect that was inhibited by AIF (Fig. 3C). To investigate the role of AIF in autophagy, we measured the expression of classical autophagy-related markers, including LC3BII, sequestosome 1 (SQSTM1/P62), and autophagy-related 5/7 (ATG5/7). Hypoxia-induced changes in expression of LC3BII, P62, ATG5 and ATG7 were reversed by AIF overexpression (Fig. 3D). Accordingly, hypoxia induced a massive flux of autophagy, with many autophagosomes (yellow) and autolysosomes (red) in PASMCs, an effect that was reversed by AIF treatment (Fig. 3E). Finally, an increased number of immature autophagic vacuoles and degradative autophagic vacuoles were displayed in the PASMCs under hypoxia, effects that were minimized by AIF overexpression (Fig. 3F). The results suggesting that hypoxia-induced mitophagy and autophagy were mainly mediated by AIF.

### AIF is involved in hypoxia-mediated PASMC proliferation in vitro and in vivo

**Fig. 4:**
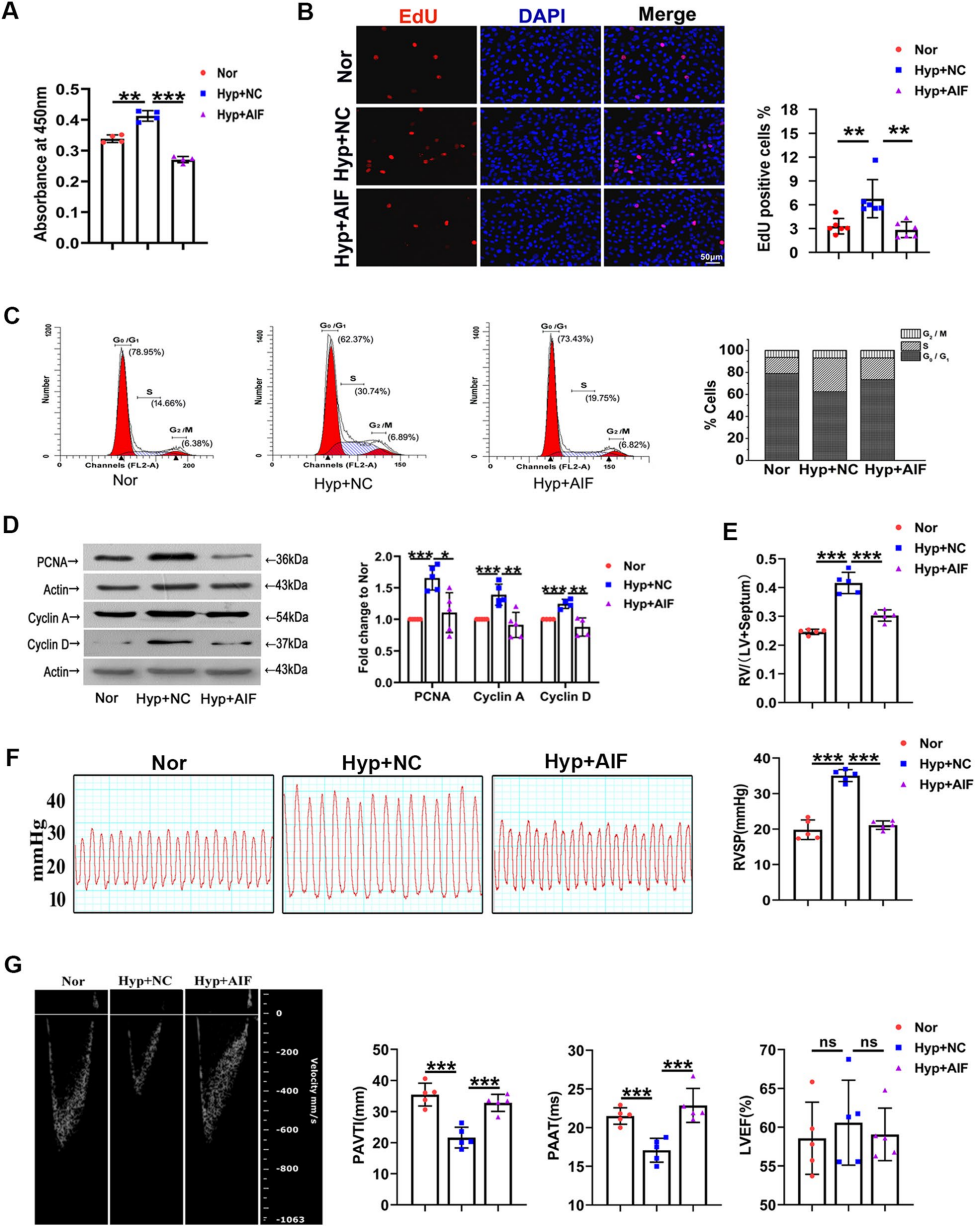
AIF blocks hypoxia-induced progression of PH in vitro and in vivo. **A** Hypoxia increased the viability of PASMCs after growth arrest for 24 h, and this effect was decreased by AIF (n = 4). **B** Pretreatment with an AIF overexpression plasmid blocked the effects of hypoxia on EdU incorporation in cells (n = 6). Scale bars: 50 μm. **C** Cell cycle analysis by flow cytometry indicated that hypoxia stimulated cell progression into G_2_/M + S phase, and this effect was inhibited by AIF overexpression (n = 3). **D** Effects of AIF on the expression of PCNA, Cyclin A and Cyclin D under hypoxia (n = 4–5). **E** Represents weight ratio of the right ventricular (RV)/left ventricular (LV) + Septum (n = 6); **F** Represents the right ventricular systolic pressure (RVSP) from mice (n = 5); **G** pulmonary artery velocity time integral (PAVTI), pulmonary artery acceleration time (PAAT) and left ventricular ejection fraction (LVEF) of the hypoxic mouse model infected with AAV5-NC and AAV5-AIF (n = 6). All data are presented as the means ± standard deviation. *p < 0.05; **p < 0.01; ***p < 0.001; *Nor* normoxia, *Hyp* hypoxia, *NC* negative control

**Fig. 5:**
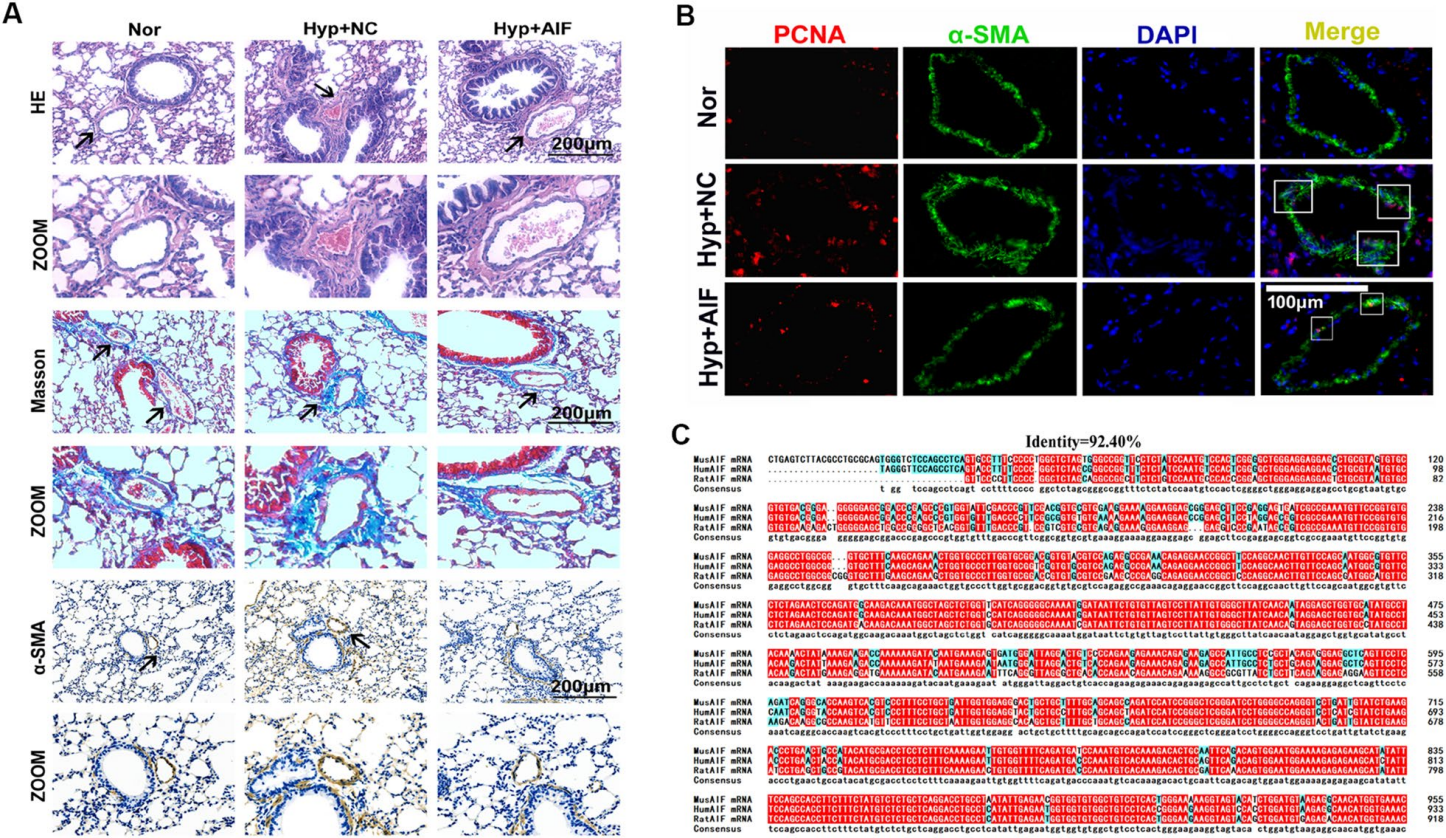
AIF blocks hypoxia-induced pulmonary vascular remodeling in vivo. **A** Morphological analysis of the pulmonary artery was performed using HE staining and Masson staining, and the thickness of pulmonary vascular medium was measured by α-SMA staining (n = 5). **B** Increased proliferation of the pulmonary vascular cells was visualized by PCNA-positive staining per vascular area under hypoxia compared with exposure to normal conditions at the same time, these effects were reversed by the administration of AAV5-AIF (n = 3). **C** Homology analysis of the AIF gene among humans, mice and rats. *Nor* normoxia, *Hyp* hypoxia, *NC* negative control

Earlier reports have implied the vital role of autophagy in cell cycle progression and the pathological progression of hypoxic PH [5]. Thus, to investigate the effect of AIF on pulmonary vascular remodeling, the cell proliferation index was first examined. Figure 4A shows that the effect of hypoxia on cell viability was reduced by AIF overexpression. We observed a similar trend in the cell proliferation of cultured PASMCs using an EdU incorporation assay (Fig. 4B). We also demonstrated that AIF is involved in hypoxia-induced expression of proliferating cell nuclear antigen (PCNA), cell cycle regulatory proteins (Cyclin A and Cyclin D) and the number of G_2_/M + S phase cells, while the effect was decreased in the presence of AIF (Fig. 4C, D). Next, to investigate the effect of AIF on pulmonary vascular remodeling in vivo, we constructed an adeno-associated virus vector 5 (AAV 5) to overexpress AIF and constructed a PH model with this vector in mice exposed to hypoxia, the overexpression efficiency of AIF in lung tissues was showed in Additional file 1: Fig. S1D. We evaluated right ventricular hypertrophy (Fig. 4E), right ventricular systolic pressure (RVSP) (Fig. 4F), echocardiography and hemodynamics (Fig. 4G) and found that AIF overexpression inhibited the pulmonary hypertension index induced by hypoxia in vivo. Furthermore, we performed pulmonary vessel morphological analysis using hematoxylin–eosin (H&E), Masson and α-smooth muscle actin (α-SMA) staining to show the potential correlations of medial thickening with AIF (Fig. 5A). Similarly, PCNA-positive staining was determined in hypoxic pulmonary arteries (Fig. 5B). The above increased indices of pulmonary vascular remodeling under hypoxic conditions were inhibited by AAV5-AIF. Additionally, Fig. 5C demonstrated that the AIF gene sequences are highly conserved among humans, mice, and rats.

### Association of AIF and UBA52

**Fig. 6:**
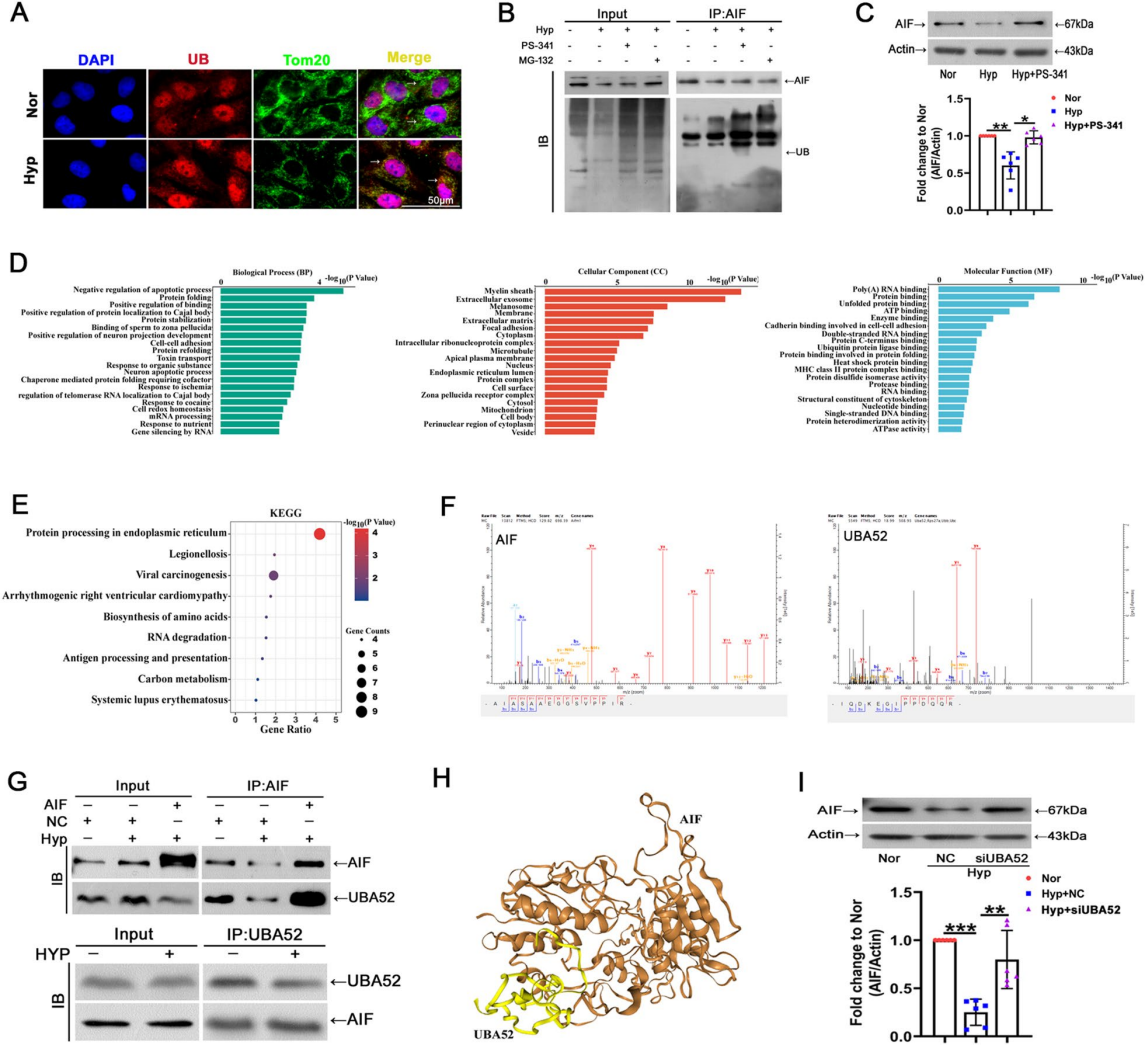
UBA52 participates in AIF ubiquitination, leading to its degradation by the proteasome system. **A** The colocalization of ubiquitin (UB) and Tom20 was determined using immunofluorescence (n = 3). Scale bars: 50 μm. **B** PASMCs were exposed to normoxia or hypoxia for 24 h, and co-IP assay was performed using anti-AIF, followed by probing with anti-UB (n = 3). **C** Cells were treated with or without PS-341 for 24 h, and the expression levels of AIF and β-actin were examined (n = 6). **D**, **E** GO and KEGG analysis of proteins interacting with AIF. **F** Mass spectrometry of specific segments of AIF and UBA52. **G** After PASMCs were exposed to normoxia or hypoxia, whole cell lysates were extracted for co-IP assay with anti-AIF or anti-UBA52, followed by probing with anti-UBA52 or anti-AIF (n = 3). **H** Representative predicted binding sites and structures of UBA52 and AIF. **I** PASMCs were transfected with si-UBA52 and then exposed to hypoxia, and the protein expression of AIF was estimated with β-actin serving as the standard (n = 6). All data are presented as the means ± standard deviation. *p < 0.05; **p < 0.01; ***p < 0.001; *Nor* normoxia, *Hyp* hypoxia, *NC* negative control, *si* small interfering RNA

Previous reports have suggested that ubiquitin induces its substrates to degrade by ubiquitination [40]. To gain a better understanding of the mechanisms underlying the reduction in AIF protein levels in PH under hypoxia, we first detected ubiquitination in PASMCs. Increased ubiquitination was shown in cells and mitochondria under hypoxia (Fig. 6A). Simultaneously, a coimmunoprecipitation (co-IP) assay was conducted using an anti-ubiquitin antibody. Ubiquitinated AIF was detected in PASMCs, while treatment with the ubiquitin-protease system inhibitors MG-132 and bortezomib (PS-341) led to the accumulation of ubiquitin proteins (Fig. 6B). Additionally, the involvement of proteasomal degradation in AIF reduction during hypoxia exposure was demonstrated by treatment with PS-341, a proteasome inhibitor, which showed partial recovery of the AIF protein levels (Fig. 6C). To identify the proteins interacting with AIF and responsible for its degradation, PASMCs were subjected to mass spectrometry using an anti-AIF antibody after immunoprecipitation. Next, we performed GO and KEGG analyses for the proteins interacting with AIF detected by mass spectrometry (Fig. 6D, E). Surprisingly, among the proteins pulled down by the anti-AIF antibody, in addition to AIF, we identified a fusion protein containing ubiquitin at the N-terminus, UBA52 (Fig. 6F). Co-IP assays further validated the presence of an interaction between UBA52 and AIF (Fig. 6G). Figure 6H shows representative molecular docking between AIF and UBA52, which was acquired from http://huanglab.phys.hust.edu.cn/. To further confirm the proteasomal degradation of AIF during UBA52 accumulation, AIF expression was examined after siUBA52. UBA52 protein expression was downregulated by transfecting the UBA52 siRNA sequence into PASMCs as shown in Additional file 1: Fig. S1B, and PASMCs treated with siUBA52 exhibited significantly increased AIF protein levels under hypoxia (Fig. 6I). These results demonstrated that the decreased AIF in hypoxia might occur by ubiquitin-mediated degradation, which is controlled by UBA52 as the ubiquitin source.

### Interaction of ubiquitinated AIF with UBA52 is required for induction of autophagy, mitochondrial dysfunction and eventually proliferation by hypoxia

**Fig. 7:**
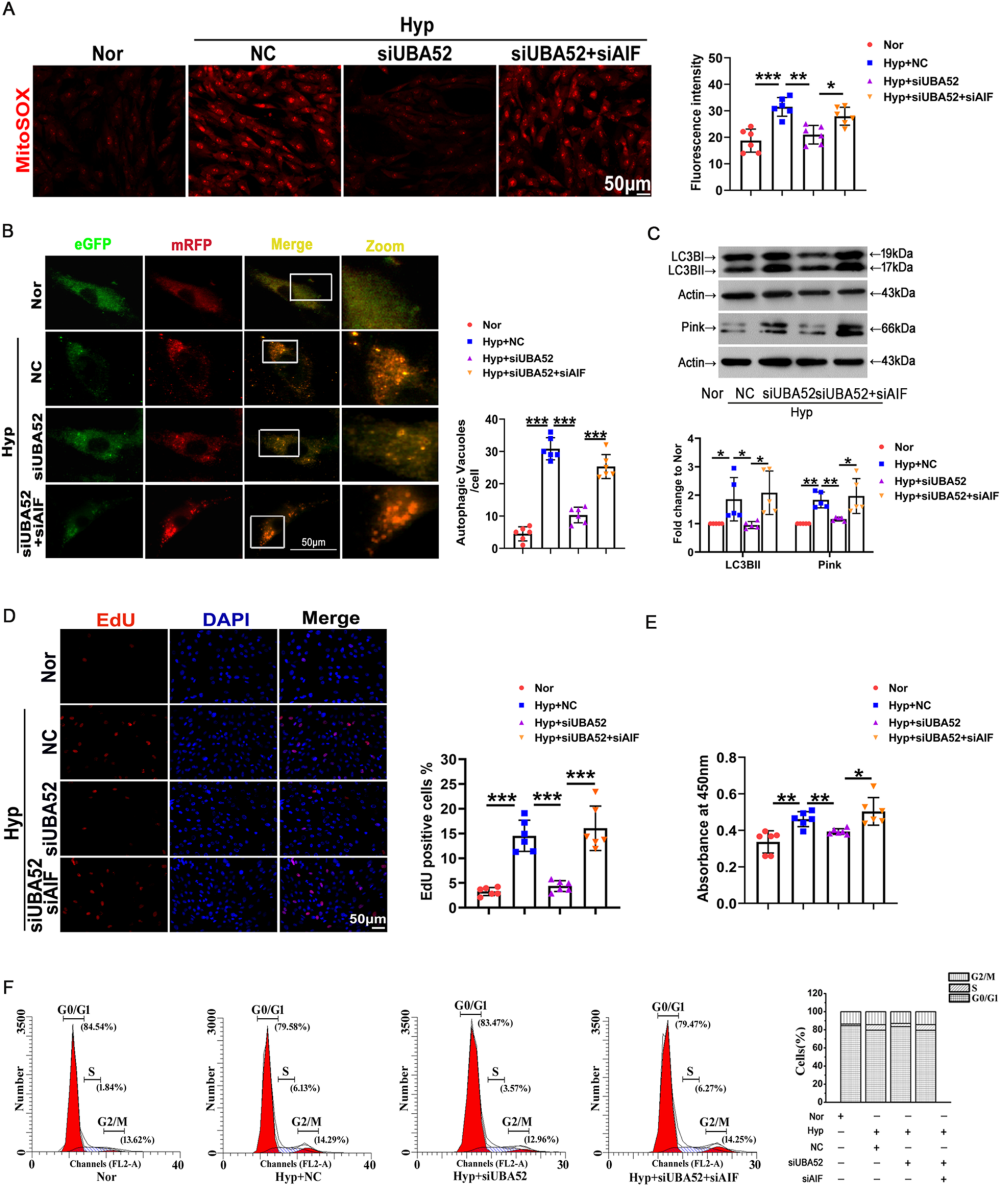
UBA52/AIF axis is responsible for increased cell autophagy and proliferation in response to hypoxia treatment. **A** Mitochondrial ROS was visualized by the mitochondrially targeted superoxide indicator (MitoSOX). Treatment with UBA52 siRNA blocked the increase in MitoSOX under hypoxia, whereas UBA52 plus AIF siRNA reversed this effect (n = 6). Scale bars: 50 μm. **B** Representative autophagic flux monitored by eGFP-mRFP LC3 plasmid transfection. The formation of autophagosomes was calculated (n = 6). Scale bars: 50 μm. **C** Western blot analysis of LC3BII and Pink in PASMCs cotransfected with UBA52 and AIF siRNA (n = 5). **D**, **E** CCK8 and 5-ethynyl-2-deoxyuridine (EdU) assays were used to determine the effects of UBA52 and AIF on cell proliferation (n = 6). **F** The number of cells in each phase of the cell cycle was examined by flow cytometry (n = 3). All data are presented as the means ± standard deviation. *p < 0.05; **p < 0.01; ***p < 0.001; *Nor* normoxia, *Hyp* hypoxia, *NC* negative control, *si* small interfering RNA

Next, we attempted to gain insight into the functional recovery experiments after deficient expression of UBA52 and AIF (the efficacy of siRNA transfection is shown in Additional file 1: Fig. S1B, C). Figure 7A shows that transfection with UBA52 siRNA reversed hypoxia-induced mitochondrial ROS release, and the effect was eliminated after siAIF. Using the eGFP-mRFPLC3 plasmid, we identified that autophagosomes were reduced by UBA52 siRNA and enhanced by siAIF cotransfection (Fig. 7B). Additionally, PASMC transfection with siUBA52 downregulated the expression of the LC3BII and Pink proteins under hypoxia, an effect that was reversed by cotransfection with AIF siRNA (Fig. 7C). EdU, CCK8 and flow cytometry assays showed that the promoting role of hypoxia in cell proliferation was relieved by the inhibition of the ubiquitination process by UBA52 siRNA treatment, whereas AIF inhibition reversed this effect (Fig. 7D–F). These results explicitly confirmed that hypoxia affected autophagy in PASMCs via the UBA52/AIF pathway.

## Discussion

AIF was identified as a caspase-independent apoptotic pathway effector that is released from mitochondria and translocates to the nucleus, where it induces chromatin condensation and DNA degradation [33, 41]. Importantly, in addition to its participation in cell death scenarios, AIF exerts a vital housekeeping function in the mitochondrial respiratory chain and redox metabolism and determines the rate of oxidative phosphorylation (OXPHOS) by regulating the stability and assembly of complex I [30, 42]. Gene silencing of AIF in HeLa cells and genetic deletion of AIF in mouse embryonic stem cells led to an impaired oxygen consumption rate and enhanced glycolysis and apoptosis resistance induced by reactive oxygen species stress [43, 44]. Mutations in the AIF gene may be responsible for a spectrum of clinical presentations, including progressive mitochondrial encephalomyopathy, muscle atrophy, auditory neuropathy and neurodegeneration [45, 46]. Based on these premises, we provide insights into new cellular consequences of the loss of AIF in PH. We showed that AIF deficiency in hypoxia triggers destabilization of mitochondrial complex I, leading to OXPHOS disorganization, high levels of mitochondrial ROS generation and increased expression of Pink and Parkin, resulting in excessive mitophagy.

**Fig. 8:**
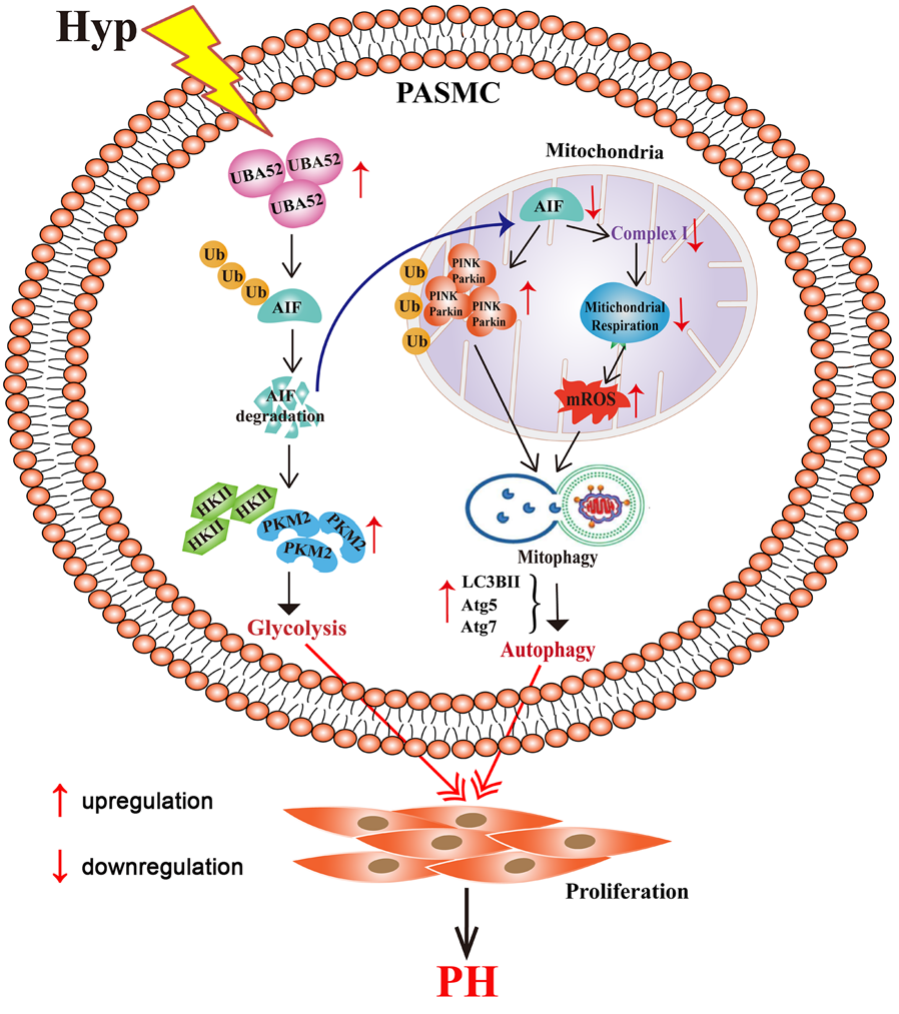
Proposed mechanism for the role of AIF in PASMC proliferation. AIF was ubiquitylated in PASMCs by interacting with UBA52 under hypoxic conditions. On the one hand, in mitochondria, the transfer of AIF to the electron transport chain is reduced, which leading to OXPHOS defects and exacerbated ROS generation-induced mitophagy and autophagy. On the other hand, in the cytoplasm, mitochondrial dysfunction together with the degradation of AIF resulted in the increased expression of HK II and PKM2 and glycolysis activation. Finally, autophagy and glycolysis regulated by AIF induce the proliferation of PASMCs

Given that mitophagy rescues cell death by clearing injured mitochondria via the autophagy machinery and that mitochondria act as signaling nodes where autophagic pathways are coordinated [47, 48], we then measured autophagy in cells with genetic overexpression of mitochondrial complex I accessory subunit AIF. Our data demonstrated that AIF overexpression inhibits the expression of ATG and LC3B proteins, reducing autophagosome formation induced by hypoxia in PASMCs. We believe that the absence of mitochondrial AIF induces a complex I and respiration damage and OXPHOS defects, and the exacerbated ROS generation by dysfunctional mitochondria ultimately improves the acute activation of autophagy by hypoxia. These results showed that AIF, as a key mitochondrial target protein, is engaged in mitophagy signaling pathways that, in turn, induces autophagy. Meanwhile, ubiquitinated and decreased cytoplasmic AIF by interaction with UBA52 in hypoxia resulted in increased expression of key glycolytic enzymes, including HK II and PKM2, indicating that the shift in mitochondrial metabolic patterns from oxidative phosphorylation to glycolysis further exacerbated PASMC proliferation (Fig. 8).

Accumulating evidence has demonstrated that ubiquitination is a common posttranslational modification that induces its substrates to degrade. The ubiquitin-coding gene UBA52 comprises ubiquitin at the amino terminus and RPL40 at the carboxy terminus and can be cleaved into RPL40 and ubiquitin [49, 50]. UBA52 is essential for preimplantation embryo development and be able to promote tumorigenesis of non-small cell lung and colorectal cancer [51, 52]. In the present study, blocking ubiquitination with PS-341 or knocking down UBA52 with siRNA significantly rescued hypoxia-mediated AIF degradation. Additionally, the results from co-IP and MS showed that UBA52 interacts with AIF, suggesting that UBA52 provides the ubiquitin pool, which serves as a crucial posttranslational modification accelerating AIF degradation by the proteasome system. Additionally, UBA52 siRNA reversed hypoxia-induced increases in mitochondrial ROS, autophagosome formation and cell cycle progression, and the effect was eliminated after cotransfection with siAIF. Thus, these results verified that the hypoxia-induced interaction between AIF and UBA52 contributed to decreased AIF and defective mitochondrial homeostasis, resulting in excessive PASMC proliferation by activating mitophagy and autophagy.

The mechanism of AIF translocation among the nucleus, mitochondria and cytoplasm along with changes in cellular function is complicated. ROS, mitochondrial permeability transition, mitochondrial fission and mitochondrial membrane potential disruption might induce the release of AIF from the mitochondria [53]. AIF shuttles from the mitochondria to the nucleus initiated by poly (ADP-ribose) polymerase 1 (PARP-1) overactivation and induces DNA fragmentation and peripheral chromatin condensation in neurotoxicity [53, 54]. AIF pathways play vital roles in both the nucleus and cytoplasm, driving excessive PASMC proliferation and survival. However, the mechanism of AIF translocation and the transport molecule are incompletely understood in hypoxic PASMCs, which would be interesting to characterize in future studies.

To summarize, we identified a hypoxia-dependent, ubiquitination-mediated mechanism for the downregulation of the key oxidoreductase in mitochondrial electron transport, AIF, in PH. In vitro functional assays and in vivo PH models confirmed that AIF overexpression inhibited PASMC autophagy and proliferation in response to hypoxia by alleviating mitochondrial dysfunction and regulating the cellular redox status. Therefore, our study suggests that AIF may be a redox-active factor essential in mitochondrial biology and can at least partially represent novel targets to develop better drugs for PASMC proliferation and PH.

## Supplementary Information

**Additional file 1: Figure S1. **A, Western blot analysis of AIF protein expression in PASMCs transfected with an AIF overexpression plasmid (n = 6). B, The interference efficiency of UBA52 was verified by Western blotting, and si3 was used in subsequent experiments (n = 6). C, Efficiency and specificity of cell-targeted siAIF. si1 and si2 similarly decreased AIF expression, and we used si2 in subsequent experiments (n = 7). D, AIF expression was significantly increased in mouse lung tissues with AAV5-AIF (n = 5). All data are presented as the means ± standard deviation. *p < 0.05; **p < 0.01; ***p < 0.001; Nor, normoxia; Hyp, hypoxia; NC, negative control; si, small RNA interfering.

## Data Availability

The datasets used and/or analyzed during the current study are available from the corresponding author on reasonable request.

## Abbreviations

PASMCs: Pulmonary artery smooth muscle cells
PH: Pulmonary hypertension
AIF: Apoptosis-inducing factor
ROS: Reactive oxygen species
RVSP: Right ventricular systolic pressure
PAVTI: Pulmonary artery velocity time integral
PAAT: Pulmonary artery acceleration time
LVEF: Left ventricular ejection fraction
RV/LV + S: Right ventricular free wall weight over sum of septum plus left ventricular free wall weight
H&E: Hematoxylin and eosin
TEM: Transmission electron microscopy
OCR: Oxygen consumption rate
ECAR: Extracellular acidification rate
CCK8: Cell counting kit 8
EdU: 5-Ethynyl-2ʹ-deoxyuridine
HK II: Hexokinase II
PKM2: Pyruvate kinase muscle isozyme 2
PDH: Pyruvate dehydrogenase
P62: Sequestosome 1
ATG5/7: Autophagy-related 5/7
PCNA: Proliferating cell nuclear antigen
AAV 5: Adeno-associated virus vector 5
α-SMA: α-Smooth muscle actin
Co-IP: Coimmunoprecipitation
