# Supplementary material for: Ubiquitinated AIF is a major mediator of hypoxia-induced mitochondrial dysfunction and pulmonary artery smooth muscle cell proliferation
Source: Cell Biosci. 2022 Jan 28;12:9. doi: 10.1186/s13578-022-00744-3 (PMC8796423; doi:10.1186/s13578-022-00744-3)
Supplement: Supplementary file 1 — Additional file 1: Figure S1. A, Western blot analysis of AIF protein expression in PASMCs transfected with an AIF overexpression plasmid (n = 6). B, The interference efficiency of UBA52 was verified by Western blotting, and si3 was used in subsequent experiments (n = 6). C, Efficiency and specificity of cell-targeted siAIF. si1 and si2 similarly decreased AIF expression, and we used si2 in subsequent experiments (n = 7). D, AIF expression was significantly increased in mouse lung tissues with AAV5-AIF (n = 5). All data are presented as the means ± standard deviation. *p < 0.05; **p < 0.01; ***p < 0.001; Nor, normoxia; Hyp, hypoxia; NC, negative control; si, small RNA interfering. [file 13578_2022_744_MOESM1_ESM.docx]

**Ubiquitinated AIF is a major mediator of hypoxia-induced mitochondrial dysfunction and pulmonary artery smooth muscle cell proliferation**


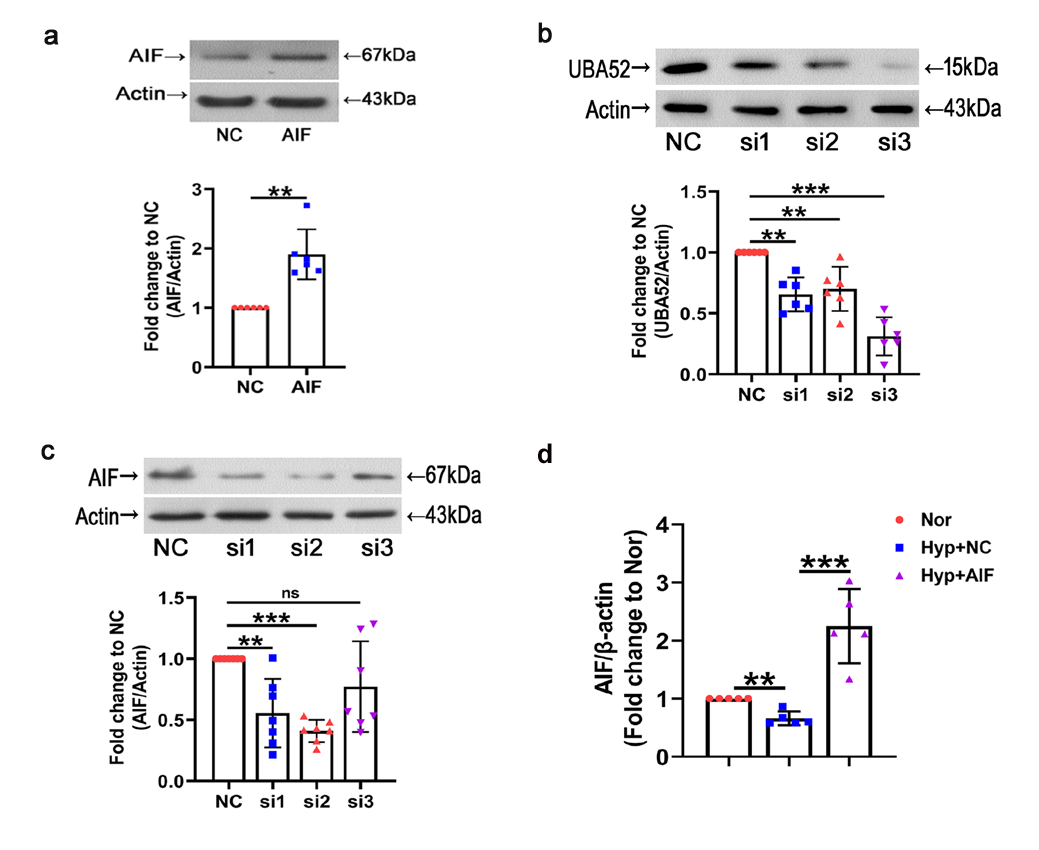


**Figure S1.** A, Western blot analysis of AIF protein expression in PASMCs transfected with an AIF overexpression plasmid (n=6). B, The interference efficiency of UBA52 was verified by Western blotting, and si3 was used in subsequent experiments (n=6). C, Efficiency and specificity of cell-targeted siAIF. si1 and si2 similarly decreased AIF expression, and we used si2 in subsequent experiments (n=7). D, AIF expression was significantly increased in mouse lung tissues with AAV5-AIF (n=5). All data are presented as the means ± standard deviation. *p < 0.05; **p < 0.01; ***p < 0.001; Nor, normoxia; Hyp, hypoxia; NC, negative control; si, small RNA interfering.
